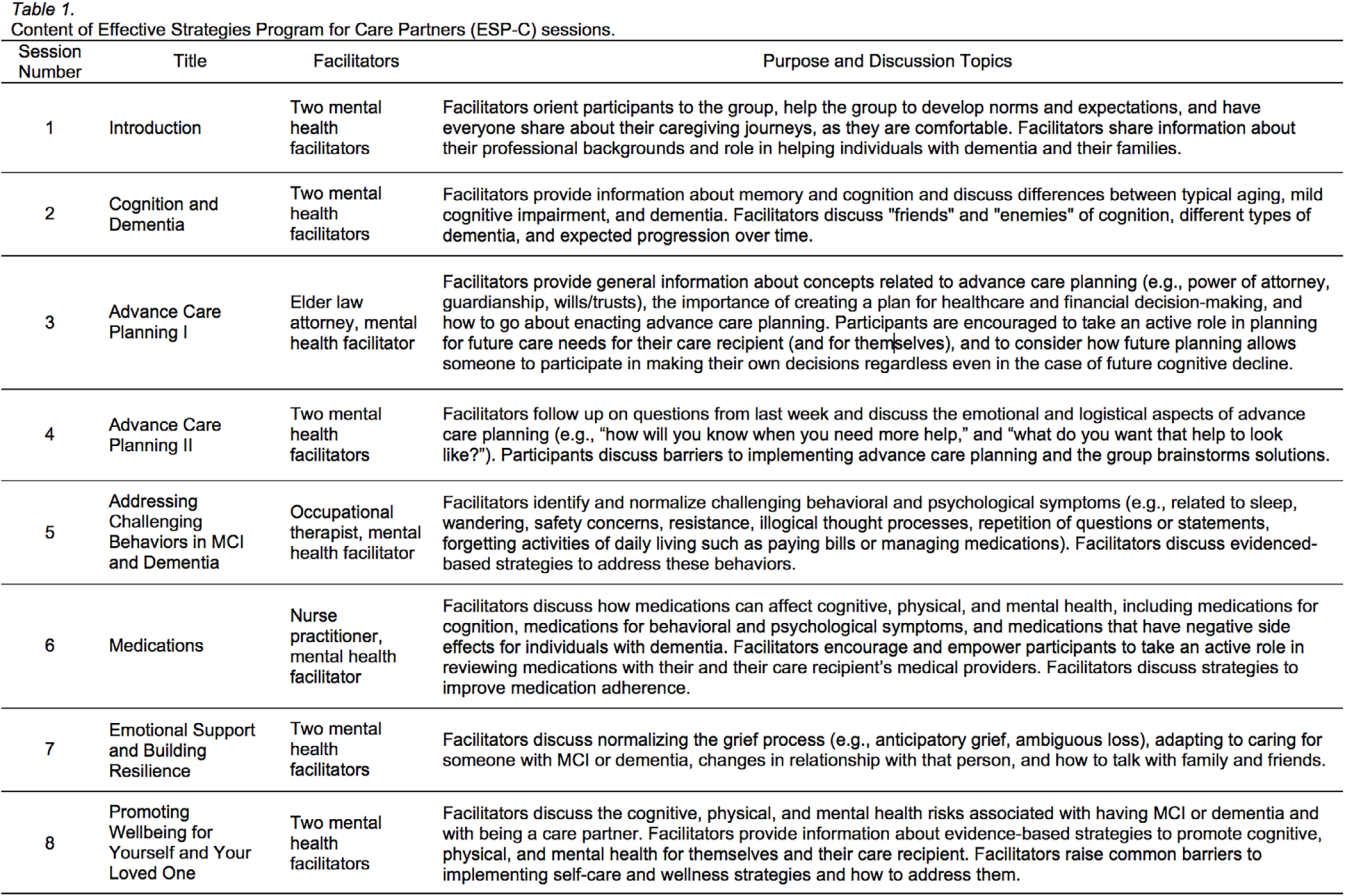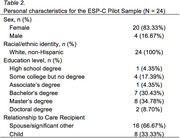# The Effective Strategies Program for Dementia Care Partners (ESP‐C): A Pilot Study

**DOI:** 10.1002/alz.086321

**Published:** 2025-01-09

**Authors:** Shannon Reilly, Virginia Gallagher, Ryan C Thompson, Ishan Canty Williams, Carol A Manning

**Affiliations:** ^1^ University of Virginia, Charlottesville, VA USA

## Abstract

**Background:**

For care of persons with dementia (PWDs), the healthcare system relies on informal care partners (CPs), who are disproportionately at risk of detrimental health outcomes. Psychosocial interventions, including via telehealth, have been shown to buffer against negative outcomes and improve CPs’ ability to provide care. We aimed to develop and pilot an evidence‐informed CP intervention using in‐person and telehealth modalities. We hypothesized that the group’s psychoeducation and socioemotional support would result in increased CP self‐efficacy and decreased burden and depression. This study examined the protocol development and initial feasibility and acceptability of ESP‐C.

**Methods:**

ESP‐C is an 8‐session supportive, psychoeducational group intervention for PWD CPs. Ninety‐minute sessions occurred weekly; see Table 1 for session topics. Groups (two in person, two via telehealth) were intended to comprise 7‐10 CPs each. Supervised care was provided for PWDs for the in‐person groups. For this pilot, we collected information about personal characteristics (Table 2), uptake, and satisfaction (e.g., “today’s session was beneficial,” “I learned something today,” “I recommend keeping this session for future groups”).

**Results:**

Twenty‐four CPs participated across 4 groups; group sizes ranged from 5‐7. Preliminary results indicate feasibility and acceptability of this program. Both session attendance (93.26%) and session satisfaction (*M* = 4.51 on a 5‐point scale) were high. Uptake of supervised care for the PWD was low (range = 0 – 2 across all sessions). Qualitative lessons learned included the benefit of adding 2 sessions (total of 8 sessions) to allow for more introduction time and discussion of advance care planning; the importance of providing resources after sessions; and that the program is not appropriate for CPs of individuals with mild cognitive impairment. The program was easily adapted for telehealth.

**Conclusions:**

This study shows initial feasibility and acceptability of ESP‐C to support PWD CPs. Importantly, the sample was relatively privileged (e.g., in terms of race, education level), underscoring the need to expand recruitment to reach underserved CPs. Next steps will include examining CP outcomes of this pilot study, as well as expanding access to the virtual ESP‐C to rural CPs without reliable internet/device access via a novel telehealth approach.